# Neural correlates of innate preference for upward motion

**DOI:** 10.1016/j.isci.2026.116352

**Published:** 2026-06-15

**Authors:** Dmitry Kobylkov, Giorgio Vallortigara

**Affiliations:** 1 Centre for Mind/Brain Science, CIMeC, University of Trento, Rovereto, Italy

**Keywords:** neuroscience, systems neuroscience, cognitive neuroscience

## Abstract

The ability to detect animate objects is a fundamental property of the animal visual system. Among the cues used to infer animacy, motion against gravity is particularly salient, as upward movement signals self-propelled, living objects. While humans and newly hatched domestic chicks show spontaneous attention to upward-moving stimuli, the neural mechanisms underlying this innate preference remain unknown. Here, we show that neurons in the nidopallium of 1-week-old domestic chicks encode upward, animacy-suggestive motion. Using extracellular recordings alongside video- and accelerometer-based behavioral tracking, we found a robust orienting response toward upward-moving stimuli. Most direction-sensitive neurons in the nidopallium responded preferentially to upward motion, and the population activity of these neurons accurately predicted the chicks’ spontaneous behavioral responses. These findings reveal a direct link between sensory encoding and innate behavioral preference, providing insight into the neural basis of sensitivity to upward, animacy-related motion cues.

## Highlights

• Chicks show spontaneous orienting to upward, animacy-suggestive motion
• Neurons in the caudal nidopallium preferentially respond to upward motion
• Neural activity predicts spontaneous behavioral responses to upward motion

## Graphical abstract

**Figure:**
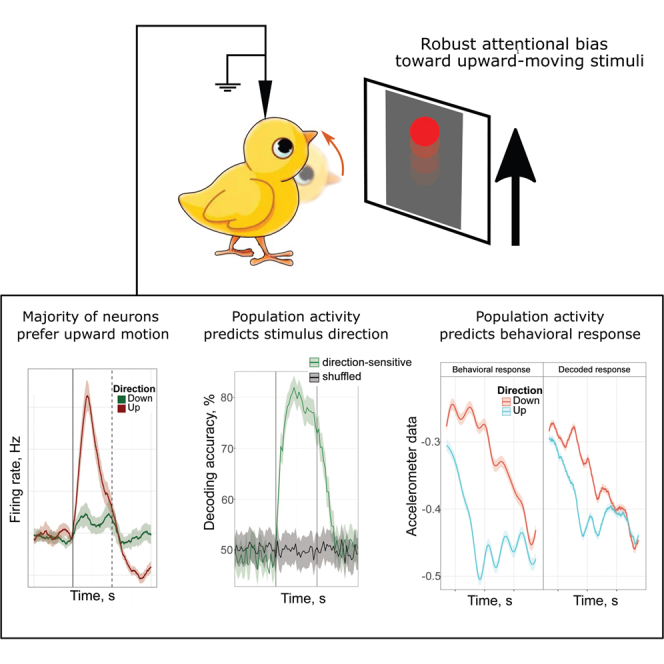

## Introduction

In an unpredictable natural environment, recognizing animate objects such as prey or predators is a core function of the visual system. Therefore, it is not surprising that the detection of certain animacy features is prewired into the brain. Soon after birth, both human infants and young domestic chicks are spontaneously attracted to animacy cues such as face-like configurations^1^^,^^2^^,^^3^ and animate motion.^4^^,^^5^

To identify animate motion, the visual system can rely on different cues, including coherent movement of body parts (biological motion^6^) or spontaneous changes in speed and direction (self-propulsion^7^). At the same time, the perception of animate motion in terrestrial organisms is strongly shaped by gravity, which constrains the biomechanics of biological motion and affects expected motion direction (e.g., inanimate objects without external force naturally fall downward). In line with this idea, biological-motion stimuli presented upside down, although retaining low-level movement coherence, become less attractive to newborn human infants.^8^ In a similar experiment, naive domestic chicks were not able to distinguish apparent movement direction of the point-light display of a walking hen (featuring coherent motion of the body joints) if it was inverted.^9^ Conversely, in astronauts, exposure to microgravity during spaceflight reduces the inversion effect.^10^

Given the pivotal role of gravity in animate-motion perception, one fundamental property of animate objects is their ability to move against gravity (i.e., upward). Indeed, humans perceive objects that move against the gravity vector as more animate.^11^ Similarly, young chicks are spontaneously attracted to upward-moving stimuli. When presented with a choice between upward- and downward-moving discs, chicks approached and spent more time near the upward-moving stimulus.^12^ At the same time, there was no difference in chicks’ responses to accelerating versus uniformly moving stimuli, indicating that gravity violation alone provides a strong trigger for animacy-related behavior.

While behavioral studies have clearly demonstrated the distinct effect of upward-moving stimuli, the neural correlates of this innate bias remain unknown. The general preference observed by Bliss et al.^12^ is a slow, long-lasting behavioral response that differs significantly from the rapid optokinetic response controlled at the subcortical level.^13^ Therefore, the perception of upward-moving stimuli is likely integrated within a broader animacy-processing network that includes higher-order brain areas. In the avian brain, one of the main candidates for this role is the caudolateral nidopallium (NCL). The NCL, a center for multimodal integration, is involved in a variety of cognitive functions, including object categorization.^14^ In a recent study, we found that neurons in the NCL of young domestic chicks selectively respond to another crucial animacy cue: face-like configurations.^15^ Moreover, the NCL has been proposed as a telencephalic area involved in global motion integration.^16^ We therefore hypothesized that the NCL might also be involved in the perception of upward, animacy-suggestive motion.

Classical behavioral paradigms used to study innate predispositions are not easily transferable to *in vivo* electrophysiological experiments. In particular, indirect behavioral measures such as time spent near a preferred object or approach latency are difficult to relate to fast neural responses. Moreover, to identify robust correlations between neural activity and behavior, multiple trial repetitions per subject are required, rather than the one-trial-per-subject design often used in behavioral research.

**Figure 1:**
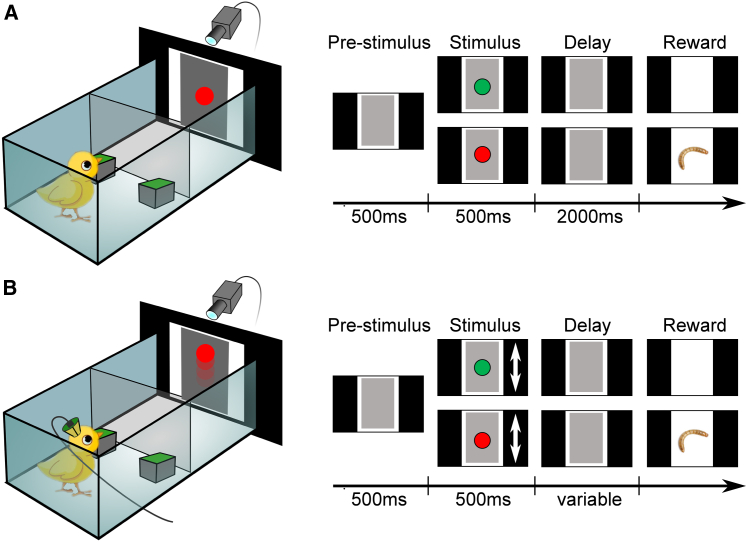
Experimental design (A) During pretraining phase, animals were presented with static discs. Red discs were subsequently rewarded, while green discs were unrewarded. (B) During the experimental phase, we recorded neural and behavioral response to upward or downward moving stimuli.

Therefore, the aims of this study were: (1) to develop a behavioral analysis pipeline capable of reliably detecting rapid spontaneous responses to intrinsically attractive upward-moving objects and (2) to identify neural correlates of the behavioral bias toward upward-moving objects in young domestic chicks. To minimize the exposure of young chicks to upward-moving stimuli, we first pretrained them to attend to the screen, where only static discs were presented (Figure 1A). Red discs were rewarded after the stimulus offset, while green discs were unrewarded. This procedure allowed us to dissociate potential reward effects in the subsequent analyses. After the pretraining period, we recorded neural activity and analyzed the spontaneous behavior of chicks as they observed upward- or downward-moving objects (Figure 1B; Video S1).

Video S1. Exemplary trials showing spontaneous behavioral response to upward motion and corresponding neural activity of a direction-sensitive neuron (A) (Left) Behavioral response shows selectivity to the upward-moving stimulus irrespective of the reward. (B) (Top right) Neural activity of an exemplary direction-sensitive unit (filtered between 300 and 6,000 Hz) in the NCL of a young domestic chick shows selective response to the upward-moving stimulus. (C) (Bottom right) Accelerometer data correspond to the upward head motion of the animal. The corresponding visual stimuli are shown in the center. The recording is slowed down to the quarter of the actual speed.

## Results

### Spontaneous response to upward moving stimuli

**Figure 2:**
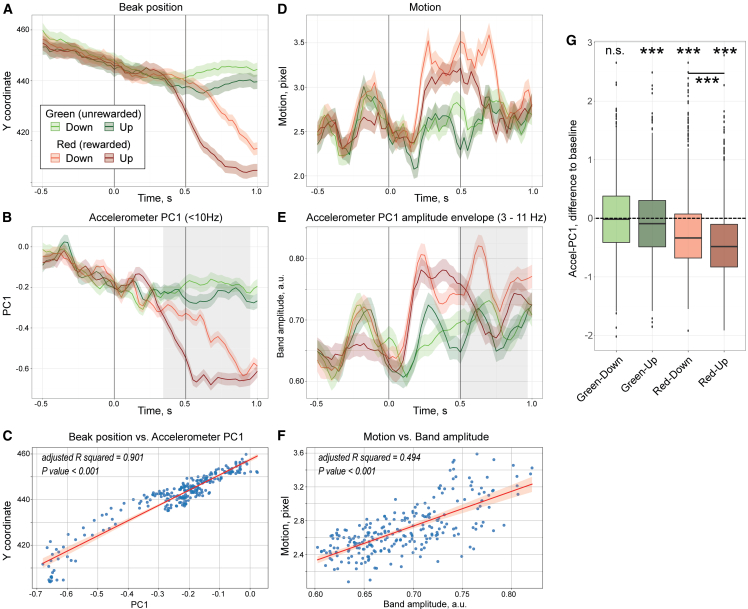
Spontaneous behavioral response to upward-moving stimuli (A) Vertical average position of the beak estimated from the video analysis with DeepLabCut. (B) Average low-frequency (<10 Hz) component of the accelerometer data (Accel-PC1). A gray-shaded area shows the time interval with a significant direction-specific response (sliding-window ANOVA: 340–950 ms after stimulus onset; permutation test: *p* < 0.001). (C) Accel-PC1 corresponds well with the beak position and therefore provides a good measure of the posture dynamics (ordinary least-squares model: *p* < 0.001, adjusted R^2^ = 0.901). (D) Overall motion response to stimuli based on the video analysis. (E) The amplitude envelope of the Accel-PC1 (in the range 3–11 Hz) corresponds to the overall motion extracted from the video. A gray-shaded area shows the time interval with a significant direction-specific response (sliding-window ANOVA: 470–950 ms after stimulus onset; permutation test: *p* < 0.001). (F) The amplitude envelope of the Accel-PC1 captures animal overall motion response (ordinary least-squares model: *p* < 0.001, adjusted R^2^ = 0.494). (G) Boxplots (median ± interquartile range (IQR), whiskers represent 1.5 ∗ IQR) show distribution of Accel-PC1 difference to the baseline, where negative values correspond to an upward head movement. Importantly, similar to the orienting response elicited by the rewarded stimulus, in response to the upward-moving stimulus chicks moved the beak upward (DeepLabCut coordinate system starts at the upper left corner). In all conditions, animals never followed downward-moving stimuli with a corresponding downward head movement. Moreover, the response to the upward-moving rewarded stimulus was significantly stronger than to the downward-moving rewarded stimulus, showing that both upward motion and reward expectation elicit an attentional bias. The Wilcoxon signed-rank test was used to compare the response to the baseline, Mann-Whitney test was used to compare “Red-Down” vs. “Red-Up” conditions. ∗∗∗ corresponds to *p* < 0.001; n.s., not significant. Shaded area along the lines in (A), (B), (D), and (E) are presented as mean ± SEM.

We first analyzed the spontaneous responses of chicks to motion stimuli by tracking the animals using markerless pose estimation with DeepLabCut.^17^ For all subsequent analyses, we included only trials in which animals viewed the stimulus with both eyes or with the contralateral (left) eye. To reveal the posture dynamics during the stimulus presentation, we calculated the average position of the beak (Figure 2A). We also used the average frame-by-frame displacement of all tracked body parts to quantify overall motion (Figure 2D).

In addition to the video analyses, we examined accelerometer data recorded alongside neural activity. To account for slight differences in accelerometer placement across individuals, we performed a principal component analysis on the three axial accelerometer channels. The first principal component (Accel-PC1) was subsequently used as a behavioral measure. We found that the low-frequency (<10 Hz) component of Accel-PC1 corresponded well with posture dynamics captured in the video analysis (Figure 2B). The average vertical position of the beak was highly correlated with the average Accel-PC1 (Figure 2C; ordinary least-squares model: *p* < 0.001, adjusted R^2^ = 0.901). Similarly, the amplitude envelope of Accel-PC1 (3–11 Hz) corresponded to the overall motion extracted from the video (Figures 2E and 2F; ordinary least-squares model: *p* < 0.001, adjusted R^2^ = 0.494). Taken together, the accelerometer data provided reliable measures of both posture and motion at a far higher temporal resolution (30 kHz) than the video tracking (∼25 Hz).

To examine how chicks spontaneously responded to upward-moving stimuli, we analyzed changes in posture (average Accel-PC1) over time across reward conditions (rewarded vs. unrewarded) and movement directions (up vs. down) using a sliding-window two-way ANOVA (100 ms window, 10 ms steps). We found that posture differed significantly between upward- and downward-moving stimuli beginning 340 ms after stimulus onset and lasting for 610 ms (Figure 2B; permutation test: *p* < 0.001). To select a time-window where the reward additionally modulated the stimulus response, we performed an additional sliding-window analysis on Accel-PC1 (<10 Hz) posture dynamics data selecting time bins significant for the interaction factor “Reward ∗ Direction”. This analysis has shown a significant window (permutation test *p* = 0.03) between 330 and 880 ms after the stimulus onset. In contrast, differences between upward and downward stimuli in the overall motion response (amplitude envelope of Accel-PC1) were significant only toward the end of the stimulus, between 470 and 950 ms after onset (Figure 2E; permutation test: *p* < 0.001). Because average Accel-PC1 (i.e., posture dynamics) showed faster and more pronounced direction-specific differences, we used this measure for subsequent analyses.

To test whether the response to downward-moving stimuli simply mirrored the response to upward-moving stimuli, we compared the average Accel-PC1 in the direction-specific window (340–950 ms) with the preceding baseline level (average in the window between 270 ms before the stimulus onset and 340 ms after the stimulus onset) for every stimulus condition (Figure 2G). Both for rewarded and unrewarded stimuli, upward moving stimuli elicited significant upward head motion (paired Wilcoxon signed-rank test with *p* values adjusted for multiple comparisons: “Red-Up”: *W* = 70320, *p* < 0.001, effect size = 0.6; “Green-Up”: *W* = 170164, *p* < 0.001, effect size = 0.13). Surprisingly, however, downward-moving stimuli in rewarded trials also resulted in a significant upward head motion (“Red-Down”: *W* = 125505, *p* < 0.001, effect size = 0.41), although this behavioral response was significantly weaker than to the upward motion for rewarded trials (“Red-Down” vs. “Red-Up”: Mann-Whitney test: *U* = 546140, *p* < 0.001). In contrast, unrewarded downward-moving stimuli did not elicit a significant response (“Green-Down”: *W* = 223625, *p* = 0.749, effect size = 0.02).

### Neural response to moving stimuli

**Figure 3:**
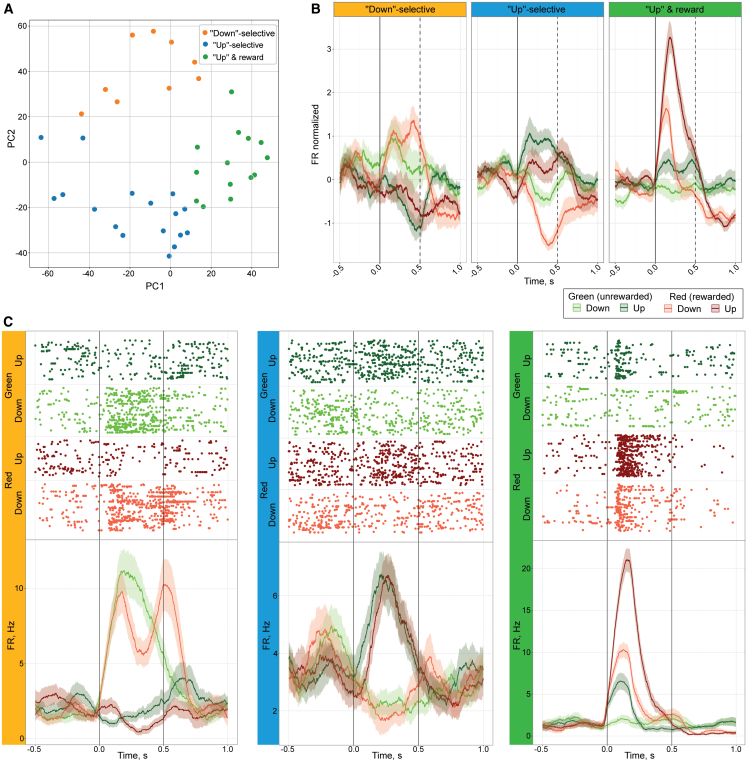
Classification of direction-sensitive neurons (A) Based on the principal component analysis of neural responses to upward/downward and rewarded/unrewarded stimuli and *k*-means clustering three groups of direction-sensitive neurons were identified (silhouette statistics for three clusters = 0.426). (B) Average response of neurons from three clusters. Neural response was Gaussian-smoothed (100 ms sigma), normalized (*Z* score) and averaged for each group. (C) Exemplary neurons from each cluster. In the raster plot (top), trials are grouped by stimulus condition and each dot corresponds to a spike. The peristimulus time-histogram (bottom) represents the average Gaussian-smoothed (100 ms sigma) neural response to stimuli. Shaded area along the lines in (B) and (C) are presented as mean ± SEM.

We recorded neural activity from 213 neurons in the NCL of young domestic chicks while they observed moving stimuli. Using a sliding-window two-way ANOVA (Gaussian-smoothed neural activity: 100 ms sigma, 10 ms steps), we found that 19% of neurons (*N* = 40) responded significantly to stimulus direction (Figures 3 and S1). There was no difference between individuals in the proportion of direction-selective neurons (*N* = 22 (19%) vs. *N* = 18 (18%), proportion test *p* = 0.887; Table S1) as well as in the relative proportion of units from different clusters (Chi-squared test: Chi^2^ = 4.09, df = 2, *p* = 0.129; Table S1).

To further characterize the responses of direction-sensitive neurons, we performed a principal component analysis on their average normalized responses across the four conditions (rewarded-up, rewarded-down, unrewarded-up, unrewarded-down). The first two components of the resulting PCA matrix were used for clustering (Figure 3A), which has been performed to describe the variability of the neural responses to a combination of stimuli factors (reward vs. motion direction). In this way, we identified three distinct clusters of neurons (the silhouette statistic for three clusters (0.426) was better than for two clusters (0.419); Figures 3B and 3C). Only 22.5% of neurons (*N* = 9) responded more strongly to downward-moving stimuli, whereas the remaining neurons preferred upward motion. The responses of 35% of neurons (*N* = 14) were modulated not only by direction but also by the reward associated with the stimuli. However, the majority of neurons (42.5%, *N* = 17) were not strongly affected by the reward and responded stronger to upward moving stimuli irrespective of the reward.

### Population response

**Figure 4:**
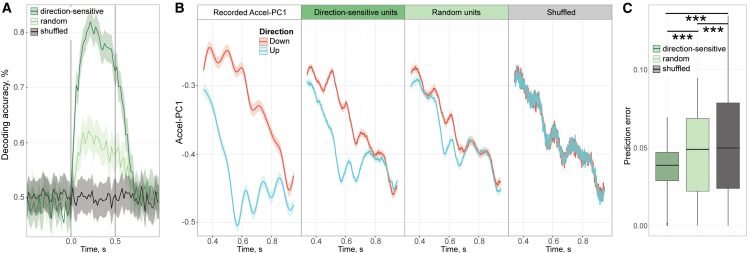
Analyses of the neural population response (A) Time-resolved decoding accuracy of SVMs trained on the neural activity of direction-sensitive units (dark-green), randomly selected units (light-green), or shuffled neural data (black). Shaded area is a 95% confidence interval. (B) Decoding of the observed behavioral response (Accel-PC1) to upward-moving stimuli with a linear regression model trained on activity of direction-sensitive units or randomly selected units. The chance level was obtained by randomly shuffling the neural data of direction-sensitive units for each time point independently to disturb the direction-dependent but not the time-dependent variability. (C) Prediction accuracy calculated as an absolute prediction error (i.e., lower error values correspond to better decoding). Boxplots represent median ± interquartile range (IQR), whiskers represent 1.5 ∗ IQR. Pairwise comparisons with permutation test: ∗∗∗ correspond to *p* < 0.001. Shaded area along the lines in (A) and (B) are presented as mean ± SEM.

To determine whether population activity could predict stimulus direction, we performed a time-resolved decoding analysis (Figure 4A). We trained support vector machines (SVMs) on the firing rate of direction-sensitive neurons in a sliding window of 200 ms (20 ms steps). Prediction accuracy was above the chance level (estimated by shuffling the training dataset) during the whole stimulus presentation period reaching over 80%. At the same time, SVMs trained on randomly selected neurons showed overall worse prediction accuracy.

Furthermore, we tested whether the activity of direction-sensitive neurons could also underlie the behavioral response to the upward-moving stimulus. For this analysis, we trained a linear regression model to decode time-varying behavioral response (Accel-PC1) from the neural activity (Figure 4B). Using the population activity of direction-sensitive neurons, we successfully decoded the behavioral response within the direction-selective window between 340 and 950 ms after the stimulus onset (Figure 4C; Kruskal-Wallis test: *H* = 179.085, *p* < 0.001). Prediction accuracy of this decoder was significantly above the chance level (pairwise comparisons with permutation test: *p* < 0.001, effect size = 0.26) and better than the decoding performed with randomly selected neurons (*p* < 0.001, effect size = 0.28). Although randomly selected units also performed above chance, the effect size was very small (*p* < 0.001, effect size = 0.06).

## Discussion

So far, studies on innate predispositions have largely relied on simplified behavioral measures of attention, such as time spent near a stimulus or approach rate.^12^^,^^18^^,^^19^ Here, we describe an approach for analyzing spontaneous behavioral responses based on both video tracking and high-speed accelerometer data (Accel-PC1). The accelerometer data allowed us not only to analyze the animal’s overall motion but also to capture its posture dynamics. We implemented this approach to describe a spontaneous response of young domestic chicks to upward-moving stimuli. Since previous behavioral experiments^12^ revealed that an upward-moving disc elicits a preference bias in newly hatched chicks, we decided to use identical upward- and downward-moving stimuli. While these simple stimuli do not reflect the full complexity of animate motion, they allowed us to test how animals spontaneously react to one of important cues for animacy detection, i.e., motion against the gravity vector.

Analyzing chicks’ posture dynamics, we found that they generally responded to attractive stimuli by moving their heads upward. This was clearly visible when comparing rewarded and unrewarded trials and can therefore be interpreted as an orienting response. However, the orienting response was significantly modulated not only by reward but also by stimulus direction. Upward head movements in response to rewarded upward-moving stimuli were significantly more pronounced than those elicited by rewarded downward-moving stimuli. Moreover, the unrewarded upward-moving stimulus also elicited a significant orienting response. At the same time, it seems unlikely that the response to upward-moving objects reflects simple object-following behavior. If this were the case, we would expect downward-moving objects to elicit a comparable response—namely, downward head movements. However, even for rewarded downward-moving objects, chicks responded with upward head movements. Apparently, both innately conspicuous upward motion and reward expectation itself elicit an arousal response (upward head movement). This effect might explain why animals respond to rewarded downward-moving stimuli with upward motion. Conversely, in the unrewarded trials, chicks did not show any significant response to downward motion. Finally, the direction-selective behavioral response persisted for almost 500 ms after the stimulus had disappeared. Together, these findings strongly suggest that upward-moving objects robustly elicit an attentional bias in chicks.

By recording neural responses in the NCL of 1-week-old chicks, we found that 19% of neurons significantly modulated their activity according to stimulus motion direction. In line with the observed behavioral bias, the vast majority of direction-sensitive neurons responded more strongly to upward motion. Moreover, cluster analysis revealed that nearly half of the direction-sensitive neurons were influenced primarily by stimulus direction rather than reward. This selective response to motion direction is consistent with previous findings showing that in the NCL of crows performing visual discrimination task 28% of all recorded cells showed direction-selectivity.^16^ Thus, the NCL appears to be an important brain center for integration of motion cues processed via both the tectofugal^20^^,^^21^ and the thalamofugal visual pathways.^22^

However, a clear bias to the upward-moving stimuli that we observed in the neural recordings in young chicks contrasts with results from other bird species. In pigeons, Frost and DiFranco^20^ showed that only 7% of motion-selective units in the tectum were tuned to the upward-moving stimuli, while the majority (37%) preferred downward motion. In the entopallium, which gets visual input from the tectum via the nucleus rotundus, the directionality tuning also remains biased toward downward-moving stimuli.^21^ Similarly, the downward bias has been observed in the visual Wulst of awake owls^22^ and in the NCL of crows.^16^ One possible explanation for these species-specific differences in direction-selective neural responses may lie in the ecological specialization of chickens. As primarily ground-dwelling animals, chickens may have different directional sensitivities compared to actively flying species. Moreover, in our experiments the chicks were not trained to discriminate motion directions, and we minimized their exposure to upward-moving objects during pretraining by presenting only static stimuli. Hence, they were far less exposed to upward-moving stimuli than the adult, trained animals used in previous studies.^16^^,^^21^ Therefore, although we cannot completely exclude the effect of learning that happens within the first week after hatching, it seems highly unlikely that the attentional bias that we observe in our 1-week-old animals has a completely different neural mechanism from those observed in newly hatched animals.^12^

Population analyses of direction-sensitive neurons in the NCL of chicks confirmed that the activity of these neurons encodes the motion direction of the stimulus. Furthermore, by training a linear regression model on the neural responses of direction-sensitive neurons, we were able to successfully decode the animal’s behavioral response. The premotor function of the NCL has been previously described in crows^23^ and pigeons.^24^ However, unlike our study, previous work focused on the motor control of voluntary movements. In crows, the sensorimotor neurons were shown to translate perceived numerical stimulus into number of actions.^23^ In pigeons, a small percentage of units in the NCL exhibited higher firing rates prior to beak movements during a Go/NoGo task.^24^ Instead, we show that NCL activity in chicks predicts spontaneous responses to innately attractive upward-moving stimuli.

Importantly, despite the strong correlation between the activity of NCL neurons and the behavioral response as revealed by our population decoding analyses, the functional role of the NCL remains ambiguous. As one of the major hubs of the avian brain NCL receiving direct projections from both the tectofugal and the thalamofugal visual pathway and the corresponding brain regions encoding motion directionality: the visual Wulst and the entopallium.^25^ This afferent connectivity makes the NCL a telencephalic site for global motion integration.^16^ At the same time, the NCL sends premotor projections to the intermediate arcopallium (AI) and the medial striatum (MSt), which makes this region also crucial for executive functions.^26^ Therefore, the NCL might be involved in perception of animacy-related features, and serve as an intermediate sensorimotor transformation stage. To further investigate the exact function role of the NCL, it would be interesting to perform experiments with targeted inactivation of this region.

In conclusion, our study provides the first insight into the neural mechanisms underlying an innate behavioral bias toward upward-moving, animacy-suggestive stimuli. By implementing a behavioral analysis pipeline combining automated video tracking and high-speed accelerometer data, we characterized spontaneous behavioral responses to upward-moving stimuli. Furthermore, we identified a neural population in the NCL of 1-week-old chicks that preferentially responds to upward-moving stimuli and encodes the animals’ posture dynamics during these responses.

### Limitations of the study

Since the main focus of our study was on the neural correlates of the bias toward upward-moving objects, we implemented only upward and downward moving stimuli not covering the full 360-degrees directionality range. Therefore, our findings are limited to differences between upward and downward motion and do not constitute evidence for general motion selectivity or animacy processing. For a comprehensive comparison with previous studies, further experiments using a wider range of motion directions and stimulus features would be required. While differences in experimental design may contribute to discrepancies with previous findings, additional work will be necessary to determine whether the observed effects are species-specific or influenced by other factors such as sex or age.

Another important factor that might have influenced our results is the rearing conditions. In our experiments, chicks were incubated in darkness, which has been shown to affect brain lateralization and alter certain behavioral responses.^27^^,^^28^ For example, chicks exposed to light during development exhibit a left-side bias when attending to a target location, but show no such bias when detouring around an obstacle. In contrast, dark-incubated chicks show no side bias when targeting an object, but display a leftward bias during detour tasks.^28^ In our experiments, the stimulus was presented at the center of the screen and moved only along the vertical axis, thereby minimizing the influence of left-right spatial biases. However, it remains possible that the attentional bias toward upward-moving stimuli observed here could be altered or even absent in light-incubated chicks. Further experiments using different incubation conditions are therefore needed.

## Resource availability

### Lead contact

Further information and requests for resources and reagents should be directed to and will be fulfilled by the lead contact, Giorgio Vallortigara (giorgio.vallortigara@unitn.it).

### Materials availability

This study did not generate new, unique reagents.

### Data and code availability

• Neural activity and behavioral tracking data have been deposited at Zenodo and are publicly available as of the date of publication (DOI: https://doi.org/10.5281/zenodo.20381425).
• All original codes for stimulus presentation and data analyses have been deposited at Zenodo at (DOI: https://doi.org/10.5281/zenodo.20381425) and is publicly available as of the date of publication.
• Any additional information required to reanalyze the data reported in this paper is available from the lead contact upon request.

## Acknowledgments

We would like to thank Mirko Zanon for his comments on the experimental design. We are also grateful to the people at the Animal House Facility for their help with handling the chicks. This study has been supported by funding from the 10.13039/501100000781European Research Council under the European Union’s Horizon 2020 research and Innovation Program No. 833504 SPANUMBRA (G.V.), PRIN 2017 ERC-SH4-A 2017PSRHPZ (G.V.), and PRIN 2022 PNRR—Grant Agreement P2022TKY7B (G.V.).

## Author contributions

D.K. and G.V. designed research; G.V. provided the funding; D.K. performed research and analyzed data; D.K. and G.V. wrote the paper.

## Declaration of interests

The authors declare no competing interests.

## STAR★Methods

### Key resources table

**Table:** 

REAGENT or RESOURCE	SOURCE	IDENTIFIER
**Deposited data**		

Raw data and codes for analyses	This manuscript^29^	https://doi.org/10.5281/zenodo.20381425

**Experimental models: Organisms/strains**		

Domestic chicken (Gallus gallus domesticus) Aviagen ROSS 308 strain	CRESCENTI Società Agricola S.r.l. –AllevamentoTrepola– cod. Allevamento127BS105/2	N/A

**Software and algorithms**		

DeepLabCut	Nath et al.^17^	URL: https://www.mackenziemathislab.org/deeplabcut
PyCharm	N/A	URL: https://www.jetbrains.com/pycharm/
Bonsai	Lopes et al.^30^	URL: https://bonsai-rx.org/
BonVision	Lopes and Monteiro^31^	URL: https://bonvision.github.io/
MountainSort4	Chung et al.^32^	URL: https://github.com/magland/mountainsort4
OpenEphys GUI	OpenEphys	URL: https://github.com/open-ephys/plugin-GUI
Phy 2.0	Cortex-lab	URL: https://github.com/cortex-lab/phy

### Experimental model and study participant details

We used two male domestic chicks (*Gallus gallus domesticus*) from the Aviagen ROSS 308 strain for this study. Fertilized eggs (CRESCENTI Società Agricola S.r.l.–AllevamentoTrepola–cod. Allevamento 127BS105/2) were incubated (Marans P140TU-P210TU) and hatched at 37.7°C and 60% humidity in darkness in the animal facility. After hatching, chicks were isolated and housed individually in metal cages (28 cm wide × 32 cm high × 40 cm deep) at a constant room temperature of 30°C–32°C and a light–dark regime of 14 h light and 10 h dark with food and water provided *ad libitum*. All experimental protocols were approved by the research ethics committee of the University of Trento and by the Italian Ministry of Health (permit number 539/2023-PR).

### Method details

#### Experimental setup

Experiments were performed in a rectangular shaped arena (34 × 54 × 27 cm; W X L X H) with wooden walls and floor covered with non-reflective materials. One wall of the arena was replaced by a computer screen (AOC AGON AG271QG4, 144 Hz) used for stimulus presentation (Figure 1A). The arena was divided in two sections by a metal grid placed 31 cm from the screen. A custom-built automatic reward system consisted of two feeders (left and right) with mealworms, whose lid was attached to a servo motor and controlled by Arduino Uno (Figure 1A). Stimulus presentation and reward were controlled using Bonsai software^30^ with the BonVision toolbox.^31^ Experiments were recorded with a frontal camera placed at the center of the screen (Imaging Source DMK 27BUR0135, Germany) at 25 frames per second.

#### Training and experimental procedure

On the second day post hatching, chicks learned to peck on mealworms. Between the third and sixth day after hatching, the chicks were habituated to the setup and trained to pay attention to the stimuli (Figure 1A).

Experimental trials consisted of four stages: 1). To prime attention, the screen turned gray 500 ms before stimulus onset; 2). Visual stimuli (discs 25 mm in diameter, 4° angular size) were presented for 500 ms. Red discs were rewarded after the stimulus offset, while green discs were unrewarded. During pretraining phase, the stimuli remain static during the presentation. After electrode implantation (8–11 days after hatching) we presented discs (25 mm, 4° angular size) moving upward or downward (angular velocity 66°/s); 3). After stimulus offset and the delay period (variable time between 2000 and 6000 ms), one of the feeders was randomly opened for 700-1000 ms; 4). The inter-stimulus interval lasted between 2500 and 3000 ms.

#### Surgery and recordings

On the seventh day after hatching, chicks were fully anesthetized using Isoflurane inhalation (1.5–2.0% gas volume, Vetflurane, 1000 mg/g, Virbac, Italy) and placed in the stereotaxic apparatus with a bar fixed at the base of the beak and tilted 45° to ear bars. Local anesthesia (Emla cream, 2.5% lidocaine +2.5% prilocaine, AstraZeneka, S.p.A.) was applied to the ears and skull skin before and after the surgery. Metal screws were placed into the skull for grounding and stabilisation of the implant. A small craniotomy was made in the skull above the NCL (1.0 mm anterior to the bregma, 4.5 mm lateral to the midline) on the right. For extracellular recordings, we used self-wired tetrodes made from formvar-insulated nichrome wire (17.78 μm diameter, A-M Systems, USA), which were gold-plated (NeuraLynx, USA) to reduce the impedance to 250–350 kOhm (controlled by nanoZ, Multi Channel Systems, Germany). Then, a 64-channel drive (Open Ephys, USA) was assembled with 16 tetrodes, implanted, and fixed with quick adhesive silicone (Kwik-Sil, World Precision Instruments, USA) and with dental cement (Henry Schein Krugg Srl, Italy).

After the surgery, the chicks were left to recover until the next day in their home cages. Between the 8th and the 11th day after hatching we recorded neural responses to moving stimuli in the NCL of chicks. After every recording session the tetrodes were manually advanced by approximately 100 μm.

Signals were recorded via a low-profile SPI headstage with an integrated 3-axis accelerometer (OEPS-6550, Open Ephys, USA). Acquisition system (Open Ephys) integrated neural data and photodiode input controlling stimulus presentation on the screen. Spike detection and sorting were automatically performed with MountainSort4,^32^ and all identified units were manually curated using Phy 2.0. Manual curation involved only removal of noise artifacts mistakenly assigned as units and merging clusters that show clear time contingency and were split into two clusters because of algorithmic mistakes.

### Quantification and statistical analysis

All statistical analyses and data visualizations were performed in Python using custom-made scripts. The results of statistical analyses described below can be found in the results section and in the corresponding figure legends.

Video recordings were analyzed using a markerless pose-estimation technique in DeepLabCut with a model trained on a subset of frames (*N* = 600) to identify “beak”, “left eye”, “right eye”, “left LED” (from headstage), “right LED”, and “headstage base”. If the contralateral (left) eye was not visible (detection likelihood probability ≥ 0.95) for at least 50% of the stimulus presentation time the trial was excluded from further analyses. In this way, we selected only trials in which birds looked with both eyes or with the contralateral (left) eye at the stimulus (3772 valid out of 5540 total trials). To describe dynamic posture changes we used the average beak position. To analyze the overall motion, we computed average displacement of all tracked body parts. First, for each body part only frames with the high tracking accuracy (likelihood probability ≥ 0.95) were selected. Then, if two consecutive frames had valid tracking (likelihood probability ≥ 0.95) of the same body part, the displacement was calculated as $\sqrt[{2}]{{\left(x_{n+1}-x_{n}\right)}^{2}+{\left(y_{n+1}-y_{n}\right)}^{2}}$, where x and y are coordinates of the body part. Finally, a median of all body parts displacement was calculated.

Accelerometer data were first transformed via principal component analysis using the same transformation matrix for all recordings to eliminate between-individual variation in the accelerometer position. The resulting first principal component (“Accel-PC1”) was used for further behavioral analyses. The low-frequency component of the Accel-PC1 (Butterworth 10 Hz low-pass filter of the 4^th^ order) was used as a measure of animal’s posture, while the amplitude envelope of the Accel-PC1 in the frequency range between 3 and 11 Hz was used as a measure of animal’s overall motion. These frequency ranges were derived from exploratory analyses based on the best regression with the video-based tracking. In general, the low-frequency range represents the gravity-based component of the data acquired by the accelerometer, which would largely correspond to vertical head position of the animal, while higher frequencies capture animal’s motion. We further evaluated how well the accelerometer measures correspond to the parameters extracted from the video by applying a linear regression model (Ordinary Least Squares) and calculating the adjusted R squared.

To reveal the difference between upward and downward moving stimuli, we performed a sliding-window analysis of variance (two-way ANOVA, 100 ms bin window, 10 ms step-size) with stimulus condition (rewarded/unrewarded) and the motion direction (up/down) as factors. Accelerometer data (average Accel-PC1 and the amplitude envelope of the Accel-PC1) were first downsampled to 1 ms time resolution. Only bins where the response was significantly different (*p* < 0.01) between upward and downward moving stimuli were selected and the longest consecutive time window was selected. A cluster permutation test was then performed to control for multiple comparisons. For this, all F-values within a significant response window were summed up (F-real) and compared to the sum of F-values resulting from the ANOVA analysis of randomly shuffled trials (F-shuffled). This procedure was repeated 1000 times, and the response window was considered truly significant only if the F-real was higher than 95% of all F-shuffled trials (corresponding to a *p* < 0.05).

To further quantify the behavioral response to moving stimuli, we compared the average response in the direction-selective window identified earlier with the preceding window of the same length served as a baseline. The difference between baseline and direction-selective window was compared with the Wilcoxon signed-rank test with *p*-values adjusted for multiple comparisons using the False Discovery Rate (FDR) Benjamini-Hochberg (BH) procedure. An absolute rank-biserial correlation was used as a measure for the effect size.

### Direction-selective neural responses

The neural activity of recorded units was analyzed in the 500 ms window starting 100 ms after stimulus onset (to account for the visual latency of NCL neurons^33^ until 100 ms after the stimulus offset. First, every trial was smoothed using a Gaussian kernel with 100 ms sigma. To identify stimulus-selective neural responses we then performed a sliding-window two-way ANOVA (10 ms bin window, 10 ms step-size) with the stimulus condition (rewarded/unrewarded) and the motion direction (up/down) as factors. Then, only bins where the response was significantly different (*p* < 0.01) between upward and downward moving stimuli were selected. If a significant response lasted for at least a 100 ms period (10 consecutive bins) we performed an additional cluster permutation test identical to the one used for accelerometer data (described above).

To further characterize direction-selective neurons we classified them into groups based on their response to different stimulus conditions. First, the neural response of every neuron was averaged by stimulus type (Rewarded-Up, Rewarded-Down, Unrewarded-Up, Unrewarded-Down), z-scored, and aligned into one row. Then, the PCA was performed on the matrix with all direction-selective neurons, and the first two components were used to define clusters of neurons (k-means clustering, N clusters = 3).

### Population response

To confirm that neural activity of direction-sensitive neurons indeed encodes the motion direction of the stimulus, we trained support vector machines (SVMs) on the firing rates of direction-sensitive units in the sliding window of 200 ms (between 600 ms before stimulus onset and 500 ms after the stimulus offset, 20 ms step). We randomly selected 72 trials per direction (Up/Down) and one trial was randomly assigned for testing. To make the neural activity comparable between units, the firing rates were z-scored using the mean and the SD of the training set only. Then, training and testing of the SVM was performed on every consecutive time bin. For a robust estimation of the decoding accuracy, we performed 1000 iterations of the SVM training and testing, randomly selecting the trials each time. The same procedure was replicated with randomly-selected units that are not direction-sensitive. To estimate the chance level we used shuffled data, where stimulus direction labels were randomly shuffled before training. The confidence interval for each time bin was calculated according to the standard method for constructing confidence intervals around a binomial proportion for large sample sizes (Wald interval^34^) as $CI=\pm 1.96*\sqrt{p*\left(1-p\right)/1000}$, where *p* is the average accuracy for the corresponding time bin.

To reveal the correspondence between the neural activity and the behavioral response to upward-moving stimuli, we performed a decoding analysis using Accel-PC1 data down sampled to 1 ms time-resolution as a behavioral output variable. Identical to the SVM analysis, 72 random trials for upward and downward conditions were selected for each unit and one of these trials was randomly assigned for testing. The neural activity was analyzed with a rolling average (200 ms window, 1 ms step) so that each Accel-PC1 value corresponds to the average firing rate in the preceding 200 ms time window. Neural data were then z-scored for each unit with the mean and standard deviation estimated for the training trials only. To combine direction-sensitive units from different recordings into one single dataset, the Accel-PC1 response was averaged between recordings and all units were combined as predictors. Finally, the linear regression model was built for each consecutive time point independently and the estimated regression coefficients were used to predict the Accel-PC1 values in the testing trials. The whole procedure was repeated 1000 times to estimate the decoding accuracy. To estimate the chance level accuracy, the correspondence between the neural activity and the Accel-PC1 was disturbed by randomly shuffling the Accel-PC1 values for each time point independently.

The decoding accuracy was evaluated by calculating an absolute error (i.e., absolute difference between average predicted and true values per time bin) and comparing the absolute error distribution between decoders trained with direction-sensitive units, randomly selected units, and shuffled data using a non-parametric Kruskal-Wallis test and a permutation test for post-hoc pairwise comparisons. In addition, an absolute rank-biserial correlation was used as a measure for the effect size.
