# Supplementary material for: Neural correlates of innate preference for upward motion
Source: iScience. 2026 Jun 15;29(7):116352. doi: 10.1016/j.isci.2026.116352 (PMC13285829; doi:10.1016/j.isci.2026.116352)
Supplement: Document S1. Figure S1 and Table S1 [file mmc1.pdf]

iScience, Volume *29*

Supplemental information

Neural correlates of innate preference for upward motion

Dmitry Kobylkov and Giorgio Vallortigara

Supplementary Information

Animal	All units	Direction-selective (% from total)	"Down"-selective (% from selective)	"Up"-selective (% from selective)	"Up" & reward (% from selective)
Chick1	115	22 (19%)	7 (32%)	10 (45%)	5 (23%)
Chick2	98	18 (18%)	2 (11%)	7 (39%)	9 (50%)
Total	213	40 (19%)	9 (23%)	17 (43%)	14 (35%)

Table S1. Summary of the recorded neurons. The proportion of direction-selective units did not differ between two animals (proportion test: $z = 0.14$, $p = 0.887$). Similarly, the relative proportion of neurons within identified clusters did not differ between individuals (Chi-squared test: Chi2 = 4.09, df = 2, p = 0.129).

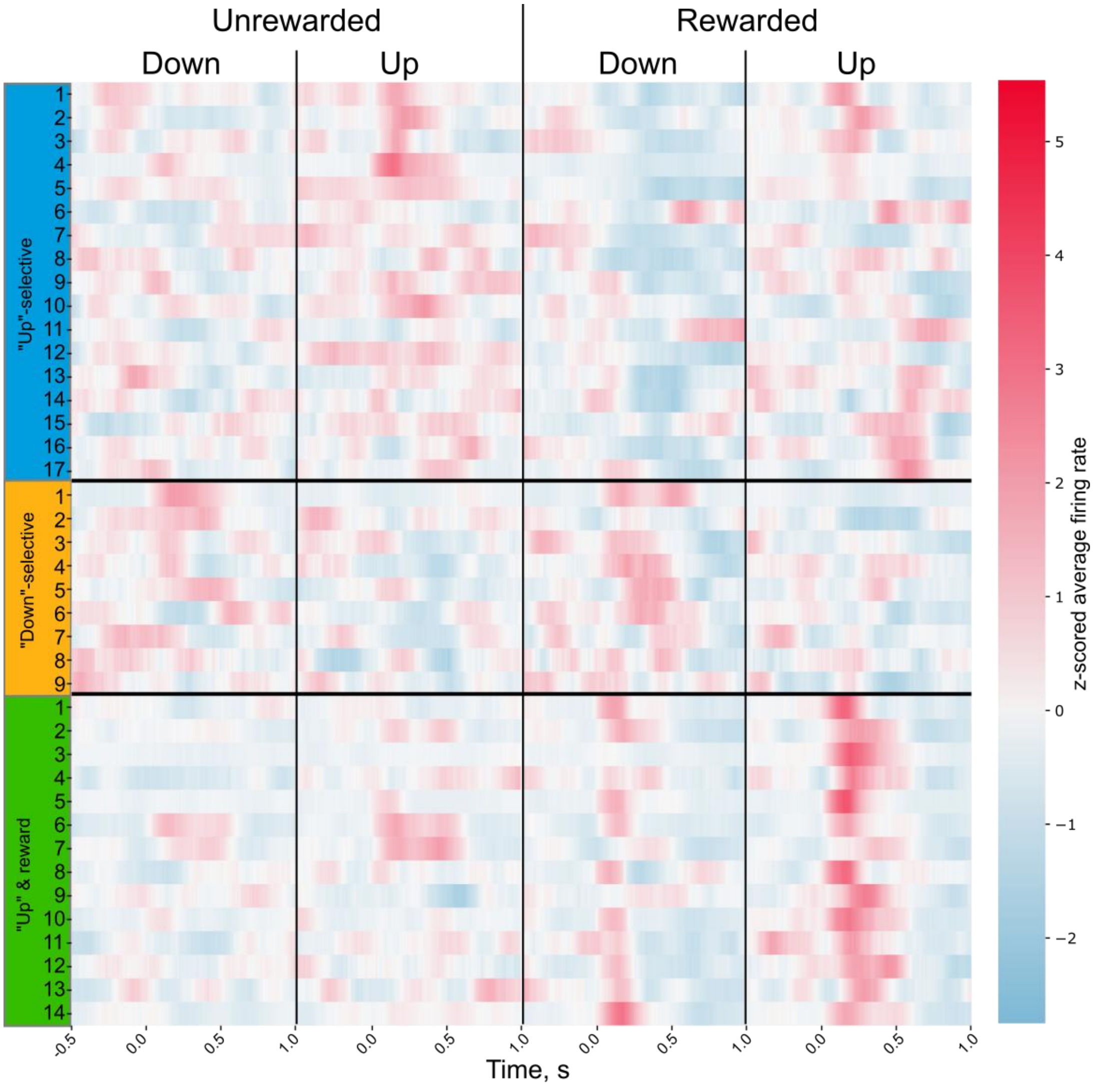

Figure S1. Heatmap showing neural responses of all direction-selective neurons. Each row represents z-scored neural activity grouped by the stimulus type. Neurons are clustered according to the PCA analysis (Fig. 3).
